# Three-dimensional characteristic of fungiform papillae and its taste buds in European bison (*Bison bonasus*), cattle (*Bos taurus*), and *Bison bonasus* hybrid

**DOI:** 10.1186/s12917-021-03111-5

**Published:** 2022-01-07

**Authors:** Barbara Plewa, Kinga Skieresz-Szewczyk, Hanna Jackowiak

**Affiliations:** Department of Histology and Embryology, Poznan University of Life Sciences, Wojska Polskiego 71C, PL 60-625 Poznań, Poland

**Keywords:** Fungiform papillae, Taste buds, Ruminants, *Bison bonasus* hybrid, Cattle, European bison, 3D- reconstruction

## Abstract

**Background:**

Our recent macro- and scanning electron microscopic study of tongue conducted on domesticated cattle, wild living European bison, and *Bison bonasus* hybrid revealed various spatial arrangement and number of gustatory and mechanical papillae between parental species and their hybrid. Furthermore, scanning electron microscopy analysis of gustatory papillae indicated the variable distribution of fungiform papillae (Fu) over the surface of the tongue, which could be significant in differentiated taste perception during feeding in studied wild living and domesticated husbandry ruminants. To specify the detailed microstructure of Fu papillae with connective tissue cores (CTC) and intraepithelial taste buds system, the first time the three-dimensional computer-aided analysis of serial histoslides resulted in the rendering of 3D reconstructions of Fu papillae.

**Results:**

The comparative analysis of 3D models Fu papillae conducted in six areas of lingual mucosa of each tongue revealed information about, microstructural diversity of Fu papillae in studied ruminants. The estimation of number and density of Fu papillae on tongues, rate of protrusion of papillae over mucosa, and a number of taste buds per papilla allowed to state the ventral surface of the lingual apex and posterolateral surfaces of the lingual torus as regions important in taste perception, as in the preselection of taken food, as well in the analysis of food during rumination, respectively. On the 3D models were observed three structural types of CTC of different distribution on the tongue in studied species. The quantitative data of the number of taste buds on Fu papillae have regional functional differences in the taste system important in feeding and veterinary practice. Moreover, our analysis determined specific features in examined hybrid and showed similarities of some studied features with cattle, i.e., maternal species.

**Conclusions:**

The 3D reconstruction method used for the first time in the field of study of the lingual papillae and taste buds system can be considered as an innovative and effective tool in assessing of the microstructures of Fu papillae, and it could be suitable for further studies of taste system structures in normal and pathological condition.

**Supplementary Information:**

The online version contains supplementary material available at 10.1186/s12917-021-03111-5.

## Background

Among lingual papillae observed on mammalian tongues, fungiform papillae (Fu papillae), vallate papillae (Vp papillae), and foliate papillae (Fo papillae) belong to gustatory papillae [1]. The occurrence of each type of gustatory papillae is characteristic in taxonomic orders of mammals. Predominantly in primates, carnivore, marsupials, marine mammals, suine, and rodents occur all three types of gustatory papillae [2–9]. However, in bats, insectivores, and some carnivores (cats) or ruminants (cattle, fallow deer, Buffalo, goat, cattle-yak, yak), only Fu papillae and Vp papillae were observed [10–24]. An interesting exception is a hippopotamus, though it belongs to herbivores, only the presence of Fu papillae and Fo papillae were described [25].

Previous macro- and microscopic studies of lingual papillae revealed that in mammalian species Fu papillae are distributed on the dorsal and ventral surface of the apex and along the body of the tongue [26]. Moreover, in ruminants these gustatory papillae are also observed on the dorsal and lateral surfaces of the lingual prominence [23, 27–30]. The distribution of Fu papillae vary on the dorsal surface of the lingual apex and body of the tongue, because Fu papillae may be located along lateral margins of the tongue or spread evenly among filiform papillae [8, 13, 27, 31, 32]. However, on the ventral surface of the tongue these papillae cover evenly lateral borders of the apex or form characteristic clusters on the border of the lingual apex or on the tip of the tongue [8, 13, 23, 31, 33, 34].

The studies on the microstructure of Fu papillae in mammals mostly based on observations in a scanning electron microscope (SEM) and histological sections in light microscopy (LM). In them Fu papillae were described as dome-shaped structures with the convex or flat dorsal surface, which raise above the mucosa or are deeply embedded in an interpapillary stratified squamous epithelium, so on SEM electronograms, these structures are encircled by epithelial furrow [3, 8, 9, 12, 21, 30, 31, 33]. In addition, Fu papillae are composed of connective tissue core (CTC) covered by keratinized stratified squamous epithelium, which contains taste buds on the dorsal surface of papilla [26, 35].

The main function of Fu papillae is taste perception by receptor cells of taste buds, which function is not only connected with the process of the choice of the food in terms of nutrients, but also with animal protection against toxins [35–40]. So far, data about the distribution and number of taste buds on the dorsal surface of Fu papillae are insufficient [4, 8, 22, 28, 31, 33, 41, 42]. Till now, it seems to be that the number of taste buds was underestimated. Neither observation of single cross-section of histoslides, nor the assessing the number of taste pores on the surface of Fu papillae, often covered by desquamated keratinized cells, was reliable. Thus, we decided to apply more specific and advanced method, like the three-dimensional analysis, to conducted studies.

Our previous microscopic studies of the tongue and mechanical and gustatory papillae showed the differences between closely related species of ruminants [27]. The differences in the distribution of Fu papillae in parental species and *Bison bonasus* hybrid were observed on both the dorsal and ventral surface of the tongue. Evenly dispersed Fu papillae on the whole dorsal surface of the apex and lingual body in *Bison bonasus* hybrid differ from the dense arrangement of these papillae on lateral areas of the lingual body in cattle and European bison. Significant diversities were also noticed on the ventral surface of the apex. In *Bison bonasus* hybrid observed V-pattern distribution of Fu papillae, whereas these papillae in cattle and European bison were arranged marginally. In turn, a similar dispersed arrangement of Fu papillae in all examined ruminants was observed on the dorsal surface of the medial and posterior part of lingual prominence and its lateral surfaces [27].

The aim of the present study was a detailed analysis of 3D models presented microstructure of Fu papillae in different areas of the tongue in three mentioned earlier species of ruminants with the histomorphometric data of the papillae. We also want proof if three-dimensional analysis will be an efficient tool in identifying many microstructures of lingual papillae as spatial relation between papillae and surrounded epithelium, the shape of internal skeleton, detailed arrangement, and a number of taste buds in Fu papillae, which will be helpful in normal, pathological, or clinical assessments.

## Results

Cross-sections of Fu papillae and 3D models with distribution of taste buds taken from particular parts of studied ruminants tongues are presented in Fig. 1.1 – 6.3. Figure 7 presents 3D models of connective tissue cores (CTCs) of Fu papillae. Table 1 contains the number and density of Fu papillae and taste buds. The values of the height and diameter of Fu papillae and connective tissue cores (CTCs) are present in Tables 2 and 3, respectively. Table 4 contains data of the height of stratified squamous epithelium on Fu papillae and the interpapillary stratified epithelium, while in Table 5 are presented the diameters of taste buds from particular parts of tongues.

**Fig. 1:**
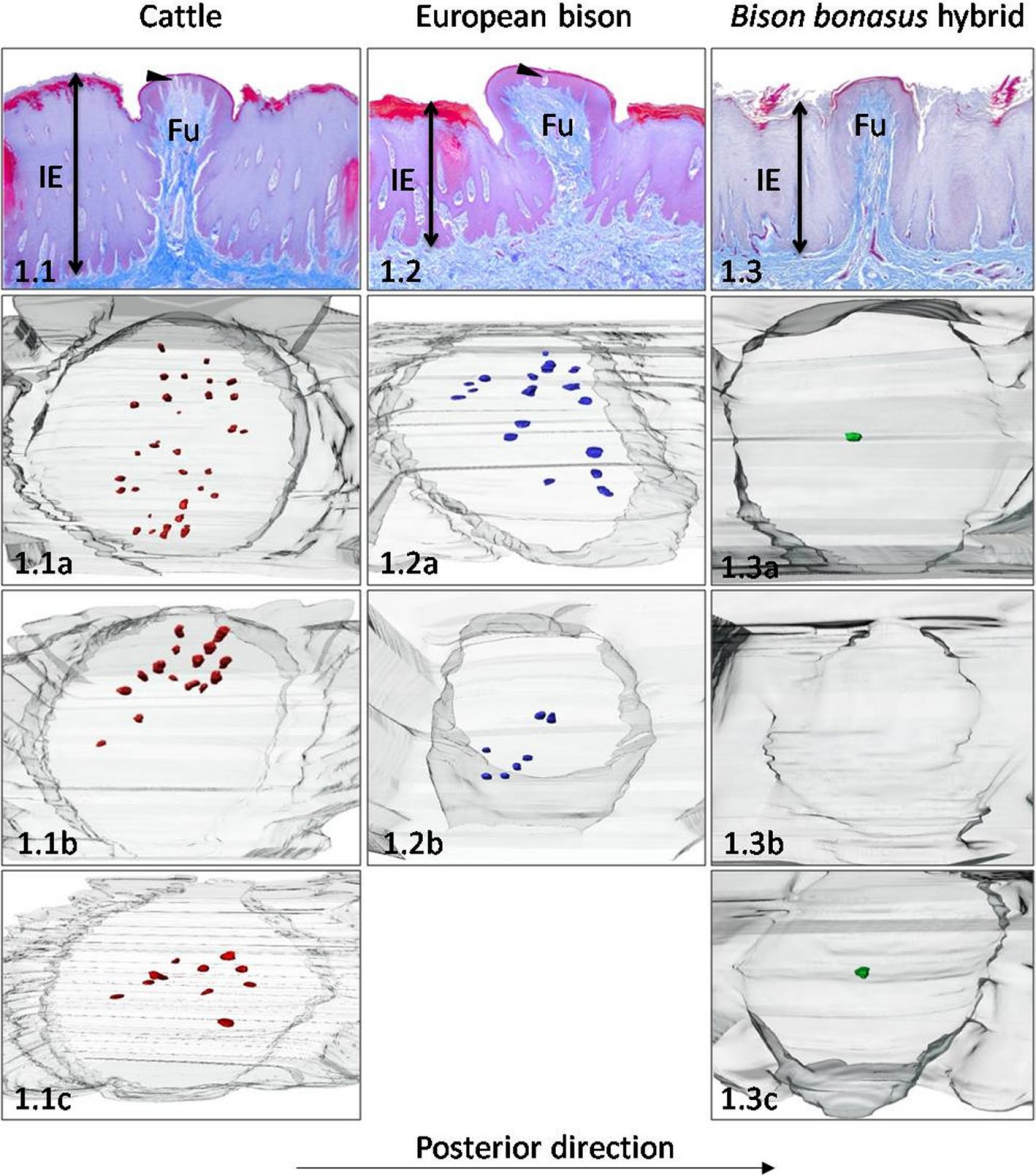
Dorsal surface of the apex. The Fu papillae and its three-dimensional reconstructions on the dorsal surface of the apex in cattle (**1.1**), European bison (**1.2**), and *Bison bonasus* hybrid (**1.3**). Fu – fungiform papillae, IE – interpapillary epithelium, black arrowhead – taste buds. Taste buds on 3D models – red dots (cattle), blue dots (European bison), green dots (*Bison bonasus* hybrid)

**Table 1:** The number and density of fungiform papillae (Fu) and its taste buds on cattle, European bison, and *Bison bonasus* hybrid tongue

	Cattle		European bison		***Bison bonasus*** hybrid	
Parts of tongue	**Number of Fu**	**Density of Fu per 1 cm**^**2**^	**Number of Fu**	**Density of Fu per 1 cm**^**2**^	**Number of Fu**	**Density of Fu per 1 cm**^**2**^
	**Number of taste buds per papilla**	**Density of taste buds per 1 mm**^**2**^	**Number of taste buds per papilla**	**Density of taste buds per 1 mm**^**2**^	**Number of taste buds per papilla**	**Density of taste buds per 1 mm**^**2**^
Dorsal surface of the apex X (Min - Max)	**13**	**0.5**	**31**	**0.4**	**222**	**9**
	**23** (10 - 31)	**20**	**12** (7 - 17)	**12**	**1** (0 - 1)	**3**
Ventral surface of the apex X (Min - Max)	**47**	**7.8**	**50**	**2.6**	**220**	**20**
	**64** (47 - 82)	**41**	**18** (6 - 30)	**20**	**20** (10 - 30)	**21**
Dorsal surface of the lingual body X (Min - Max)	**35**	**0.8**	**32**	**1**	**118**	**3**
	**41** (20 - 55)	**22**	**10** (5 - 13)	**10**	**3** (3 - 4)	**5**
Dorsal surface of medial part of torus X (Min - Max)	**15**	**0.5**	**23**	**0.8**	**15**	**0.6**
	**13** (4 - 22)	**5**	**4** (3 - 4)	**2**	**13** (5 - 20)	**11**
Dorsal surface of posterior part of torus X (Min - Max)	**30**	**0.9**	**20**	**0.9**	**46**	**2.7**
	**37** (31 - 43)	**24**	**14** (4 - 24)	**4**	**13** (8 - 18)	**11**
Lateral surfaces of torus X (Min - Max)	**45**	**1.0**	**46**	**1.1**	**38**	**1.2**
	**17** (4 - 29)	**16**	**14** (6 - 21)	**18**	**23** (16 - 29)	**19**

**Table 2:** Histomorphometry of Fu papillae on cattle, European bison, and *Bison bonasus* hybrid tongue

Parts of tongue	Fungiform papillae	Cattle	European bison	***Bison bonasus*** hybrid
Dorsal surface of the apex X (Min – Max)	Height of the papilla (μm)	**1980.7** (1756.4 – 2354.8)	**1442.2** (1219.3 – 1649.4)	**1326.2** (1200 - 1406.4)
	Diameter of the papilla (μm)	**1070.2** (759.7 – 1322.5)	**953.8** (844.4 – 1032.5)	**673.0** (554.8 – 722.6)
Ventral surface of the apex X (Min – Max)	Height of the papilla (μm)	**1358.6** (1219.3 – 1451.6)	**1016.4** (754.8 – 1205.1)	**1104** (673.1 – 1406.4)
	Diameter of the papilla (μm)	**1056.2** (903.2 – 1131.7)	**990.4** (522.6 – 1212.9)	**664.9** (490.3 – 844.1)
Dorsal surface of lingual body X (Min – Max)	Height of the papilla (μm)	**1343.9** (1187.1 – 1438.7)	**1508.2** (1187.0 – 1438.7)	**1322.7** (1269.2 – 1380.6)
	Diameter of the papilla (μm)	**1173.1** (909.6 – 1348.3)	**1102.5** (909.6 – 1348.3)	**828.9** (687.9 – 909.6)
Dorsal surface of medial part of torus X (Min – Max)	Height of the papilla (μm)	**1319.8** (1149.3 – 1435.0)	**1165.8** (612.9 – 1435.2)	**2060.1** (1902.2 – 2214.9)
	Diameter of the papilla (μm)	**1865.6** (1361.3 – 2155.8)	**1628.0** (1167.7 – 2174.2)	**1357.7** (1103.9 – 1593.6)
Dorsal surface of posterior part of torus X (Min – Max)	Height of the papilla (μm)	**1402.1** (1013.0 – 1629.8)	**1397.4** (1032.4 – 1717.9)	**1221.5** (1032.4 – 1425.8)
	Diameter of the papilla (μm)	**1095.7** (954.2 – 1313.7)	**1675.4** (1134.6 – 1883.1)	**895.0** (792.2 – 1006.4)
Lateral surfaces of torus X (Min – Max)	Height of the papilla (μm)	**1314.0** (1078.6 – 1649.3)	**1589.0** (1103.2 – 1717.9)	**1601.9** (1361.3 – 1771.2)
	Diameter of the papilla (μm)	**1071.7** (594.8 – 1461.0)	**655.6** (564.1 – 1461.0)	**964.0** (764.7 – 1214.3)

**Table 3:** Diameters of CTCs in dorsal, medial and basal part of Fu papillae on cattle, European bison, and *Bison bonasus* hybrid tongue

Parts of tongue	Diameter of CTC	Cattle	European bison	*Bison bonasus* hybrid
Dorsal surface of the apex X (Min - Max)	Dorsal part	**637.1** (445.2 – 851.6)	**635.9** (559.2 – 743.4)	**367.9** (270.9 – 451.6)
	Medial part	**358.9** (200 – 438.7)	**402.4** (322,4 – 447.4)	**334.8** (232.2 – 458.1)
	Basal part	**1013.5** (506.4 – 1303.2)	**715.0** (638.7 – 864.5)	**659.2** (425.8 – 870.9)
Ventral surface of the apex X (Min - Max)	Dorsal part	**923.8** (677.5 – 1147.7)	**571.4** (331.2 – 832.3)	**481.3** (327.8 – 600.8)
	Medial part	**620.1** (374.2 – 862.3)	**518.3** (383.2 – 766.2)	**416.2** (339.9 – 483.9)
	Basal part	**944.1** (793.5 – 1090.3)	**802.5** (519.4 – 935.5)	**682.3** (522.6 – 883.1)
Dorsal surface of the lingual body X (Min - Max)	Dorsal part	**700.4** (599.9 – 870.9)	**476.5** (387.1 – 664.5)	**603.9** (558.4 – 662.4)
	Medial part	**484.6** (400 – 625.8)	**451.9** (290.3 – 611.8)	**462.4** (337.6 – 513)
	Basal part	**1028.9** (904.4 – 1212.9)	**900.1** (904.4 -1212.9)	**768.5** (643.3 – 870.9)
Dorsal surface of medial part of torus X (Min - Max)	Dorsal part	**1404.8** (1162.4 – 1662.4)	**818.1** (583.4 - 1329)	**997.3** (724.4 – 1188,2)
	Medial part	**1698.1** (1305.2 – 1909.1)	**929.3** (524.6 – 1554.8)	**687.6** (429.7 – 831.1)
	Basal part	**1898.5** (1303.6 – 2207.8)	**1402.3** (1174.2 – 2180.6)	**1277.9** (1038.9 – 1428.6)
Doral surface of posterior part of torus X (Min - Max)	Dorsal part	**881.9** (629.9 – 1071.7)	**1439.4** (910.3 – 1764.8)	**662.2** (516.5 – 807.9)
	Medial part	**649.9** (389.8 – 831.2)	**1456.2** (967.3 – 1826.9)	**558.1** (398.7 – 642.5)
	Basal part	**1265.8** (830.9 – 1548.9)	**1769.2** (1346.1 – 2097.4)	**1067.4** (883.8 – 1181.8)
Lateral surfaces of torus X (Min - Max)	Dorsal part	**574.6** (290.4 – 961.6)	**381.8** (294.3 – 474.1)	**708.9** (483.8 – 929.5)
	Medial part	**633.2** (407.3 – 787.1)	**389.8** (193.5 – 551.9)	**570.6** (367.8 – 801.3)
	Basal part	**1056.6** (712.4 – 1467.5)	**665.9** (583.3 – 1109.7)	**1063.7** (767.7 - 1400)

**Table 4:** The histomorphometry of interpapillary stratified epithelium and stratified squamous epithelium with keratinized layer on the dorsal surface of Fu papillae in cattle, European bison, and *Bison bonasus* hybrid

Parts of tongue	Fungiform papillae	Cattle	European bison	*Bison bonasus* hybrid
Dorsal surface of the apex X (Min – Max)	Height of stratified squamous epithelium (μm)	**115.4** (13.0 – 232.6)	**140.8** (56.7 – 286.9)	**119.7** (47.6 – 217.3)
	Height of the keratinized layer of the epithelium [μm]	**17.9** (10.8 – 32.1)	**20.8** (11.5 – 63.5)	**19.2** (5.8 – 40.3)
	Height of interpapillary epithelium (μm)	**1567.3** (1506.4 – 2341.9)	**1051.7** (741.9 – 1253.9)	**1166.2** (987.1 – 1316.1)
Ventral surface of the apex X (Min – Max)	Height of stratified squamous epithelium (μm)	**108.2** (48.4 – 214.5)	**139.5** (65.3 – 264.9)	**96.9** (31.6 – 265.5)
	Height of keratinized layer of epithelium (μm)	**11.1** (4.8 – 22.4)	**14.9** (5.1 – 33.9)	**9.2** (3.6 – 27.6)
	Height of interpapillary epithelium (μm)	**1016.4** (726.5 – 1378.0)	**770.8** (309.6 – 1149.3)	**930.1** (593.5 – 1167.7)
Dorsal surface of lingual body X (Min – Max)	Height of stratified squamous epithelium (μm)	**122.3** (40.4 – 304.3)	**166.5** (67.7 – 276.9)	**118.6** (41.3 – 188.4)
	Height of keratinized layer of epithelium (μm)	**16.2** (6.6 – 30.4)	**20.6** (9.6 – 32.6)	**19.0** (8.2 – 42.2)
	Height of interpapillary epithelium (μm)	**1096.9** (519.2 – 1374.2)	**1067.1** (519.2 – 1372.2)	**1221.5** (1154.8 – 1296.8)
Dorsal surface of medial part of torus X (Min – Max)	Height of stratified squamous epithelium (μm)	**143.2** (21.9 – 302.2)	**150.6** (64.5 – 265.2)	**121.9** (31.9 – 259.9)
	Height of keratinized layer of epithelium (μm)	**29.2** (15.3 – 83.6)	**37.1** (21.0 – 65.8)	**35.8** (12.9 – 98.1)
	Height of interpapillary epithelium (μm)	**1006.9** (675.3 – 1432.2)	**838.8** (296.7 – 1432.2)	**1897.8** (1612.9 – 2148.4)
Dorsal surface of posterior part of torus X (Min – Max)	Height of stratified squamous epithelium (μm)	**112.9** (47.4 – 193.1)	**129.5** (52.2 – 259.9)	**113.3** (31.2 – 211.2)
	Height of keratinized layer of epithelium (μm)	**17.2** (6.7 – 39.2)	**19.2** (9.8 – 37.4)	**16.8** (8.1 – 32.1)
	Height of interpapillary epithelium (μm)	**470.0** (331.2 – 640.5)	**680.7** (397.4 – 948.7)	**944.4** (616.9 – 1206.3)
Lateral surfaces of torus X (Min – Max)	Height of stratified squamous epithelium (μm)	**147.1** (56.8 – 361.9)	**120.9** (50.9 – 236.9)	**121.2** (34.3 – 216.9)
	Height of keratinized layer of epithelium (μm)	**31.5** (14.8 – 74.3)	**20.9** (9.4 – 68.1)	**17.7** (5.8 – 41.9)
	Height of interpapillary epithelium (μm)	**1205.3** (864.5 – 1577.9)	**1147.8** (864.5 – 1423.0)	**1408.9** (1181.8 – 1535.5)

**Table 5:** The diameter of taste buds in cattle, European bison, and *Bison bonasus* hybrid

	Cattle	European bison	*Bison bonasus* hybrid
Parts of tongue	**Diameter of taste buds** (μm) **X** (Min - Max)	**Diameter of taste buds** (μm) **X** (Min - Max)	**Diameter of taste buds** (μm) **X** (Min - Max)
Dorsal surface of the apex	**40.9** (29.9 – 62.9)	**47.9** (38.7 – 57.5)	**32.5** (30.2 – 32.5)
Ventral surface of the apex	**39.5** (31.0 – 51.9)	**41.7** (29.6 – 52.5)	**42.6** (29.6 – 63.9)
Dorsal surface of the lingual body	**41.2** (30.2 – 55.6)	**42.4** (29.0 – 54.8)	**45.2** (39.5 – 56.4)
Dorsal surface of medial part of torus	**41.1** (37.1 – 45.7)	**45.1** (30.6 – 58.0)	**42.2** (25.9 – 56.5)
Dorsal surface of posterior part of torus	**40.6** (30.1 – 49.4)	**42.9** (29.9 – 56.0)	**36.5** (25.8 – 60.1)
Lateral surfaces of torus	**44.8** (32.2 – 63.0)	**38.8** (23.8 – 56.1)	**36.9** (24.2 – 50.3)

### Dorsal surface of the apex of the tongue

In cattle, 13 Fu papillae with the density of 0.5 papilla per 1 cm^2^ were spread regularly between filiform papillae on the dorsal surface of the apex (Table 1). The Fu papillae were round in outline with convex, smooth surface, and the diameter was 1070 μm on average (Table 2). The mean height of these papillae was ca. 1980.7 μm (Table 2, Fig. 1.1), and concerning mucosal epithelium, these papillae protruded on 413.4 μm (Table 4, Fig. 8A). Based on measurements of diameter in basal, medial, and dorsal parts of internal connective tissue of Fu papillae, the shape of CTC was described as balloon-like (Table 3, Figs. 1.1, 7A and 8B). The height of the stratified epithelium on Fu papillae reached 115.4 μm, while its keratinized layer represented 15.5% of the epithelium thickness (Table 4, Fig. 8C). Three studied 3D models of Fu papillae showed different patterns of distribution of taste buds on the dorsal surface of papillae. Taste buds were arranged in the median part of the papilla in the form of strip located perpendicularly to the median line of the tongue (Fig. 1.1a). On the other hand, taste buds formed clusters were positioned centrally or on the left side of Fu papillae (Fig. 1.1b, 1.1c). The average number of taste buds was 23, while the density reached 20 taste buds/mm^2^ of papilla surface (Table 1). Moreover, the average diameter of taste buds ranged between 29.9 μm – 62.9 μm (Table 5).

In European bison, Fu papillae were like cattle, spread evenly among filiform papillae, but its number and density reached 31, 0.4 papilla/cm^2^, respectively (Table 1). The elliptical in outline papillae have a convex and smooth dorsal surface with diameter of 953.8 μm (Fig. 1.2, Table 2). The mean height of Fu papillae reached 1442.2 μm (Table 2), whereas the protrusion of Fu papillae over the surface of lingual mucosa was 390.5 μm (Table 4, Fig. 8A). The CTC with a broad dorsal part had a balloon-like shape (Table 3, Figs. 1.2, 7A and 8B). The height of stratified squamous epithelium was 140.8 μm, and its keratinized layer was 14.7% of its thickness (Table 4, Fig. 8C). The 3D models of Fu papillae in European bison presented in average 12 taste buds arranged in clusters on the right or left part of the papilla (Table 1, Fig. 1.2a, 1.2b). The density of taste buds on the dorsal surface of Fu papillae was 12 taste buds/mm^2^ (Table 1), while the mean diameter of taste buds reached 47.9 μm (Table 5).

The *Bison bonasus* hybrid has numerous i.e., 222 evenly spread Fu papillae on the dorsal surface apex (Table 1). Their density reached nine papillae/cm^2^ (Table 1). These rounded in outline papillae were characterized by flat and smooth surfaces with the mean values of its diameter ranged between 554 μm and 722 μm (Table 2, Fig. 1.3). The difference between the height of Fu papillae – 1326.2 μm, and the height of interpapillary stratified epithelium – 1166.2 μm i.e. the value of the papillary protrusion equals 160 μm (Tables 2 and 4, Fig. 8A). Diameters of Fu papillae CTCs indicated the columnar-like shape of this internal skeleton of Fu papillae. (Table 3, Figs. 1.3, 7B and 8B). The thickness of the epithelium covering Fu papillae was 119.7 μm on average, whereas its keratinized layer amount to 16% of the epithelium thickness (Table 4, Fig. 8C). In two 3D models of these papillae, only a single taste bud of the mean diameter 32.5 μm were positioned centrally on surface of papilla (Table 5, Fig. 1.3a, 1.3c). In turn, in the third prepared model observed a lack of taste buds in dorsal epithelium (Fig. 1.3b). The mean density of taste buds was three taste buds/mm^2^ (Table 1).

### Ventral surface of the apex of the tongue

In cattle, 47 Fu papillae appeared symmetrically on lateral borders of the ventral surface of the tongue, while the mean density was 7.8 papillae/cm^2^ (Table 1). Convex Fu papillae with an average diameter of 1056.2 μm were round in outline (Table 2, Fig. 2.1). Fu papillae and interpapillary epithelium height were 1358.6 μm, and 1016 μm, respectively (Tables 2, 4). Thus, the average protrusion of these papillae was 342.2 μm (Fig. 8A). The shape of CTC of Fu papillae presented in Fig. 7A, resembled a balloon (Table 3, Figs. 2.1, 8B). The epithelium on the Fu papillae was 108.2 μm in thickness with the keratinized layer, which was 10% of its entire thickness (Table 4, Fig. 8C). In cattle, the 64 taste buds on average, were evenly arranged posteriorly or on the whole surface of the dorsal part of Fu papillae (Table 1, Fig. 2.1a, 2.1b). The density of taste buds in these papillae reached 41 taste buds/mm^2^, while the mean diameter of taste buds was 39.5 μm (Tables 1 and 5).

**Fig. 2:**
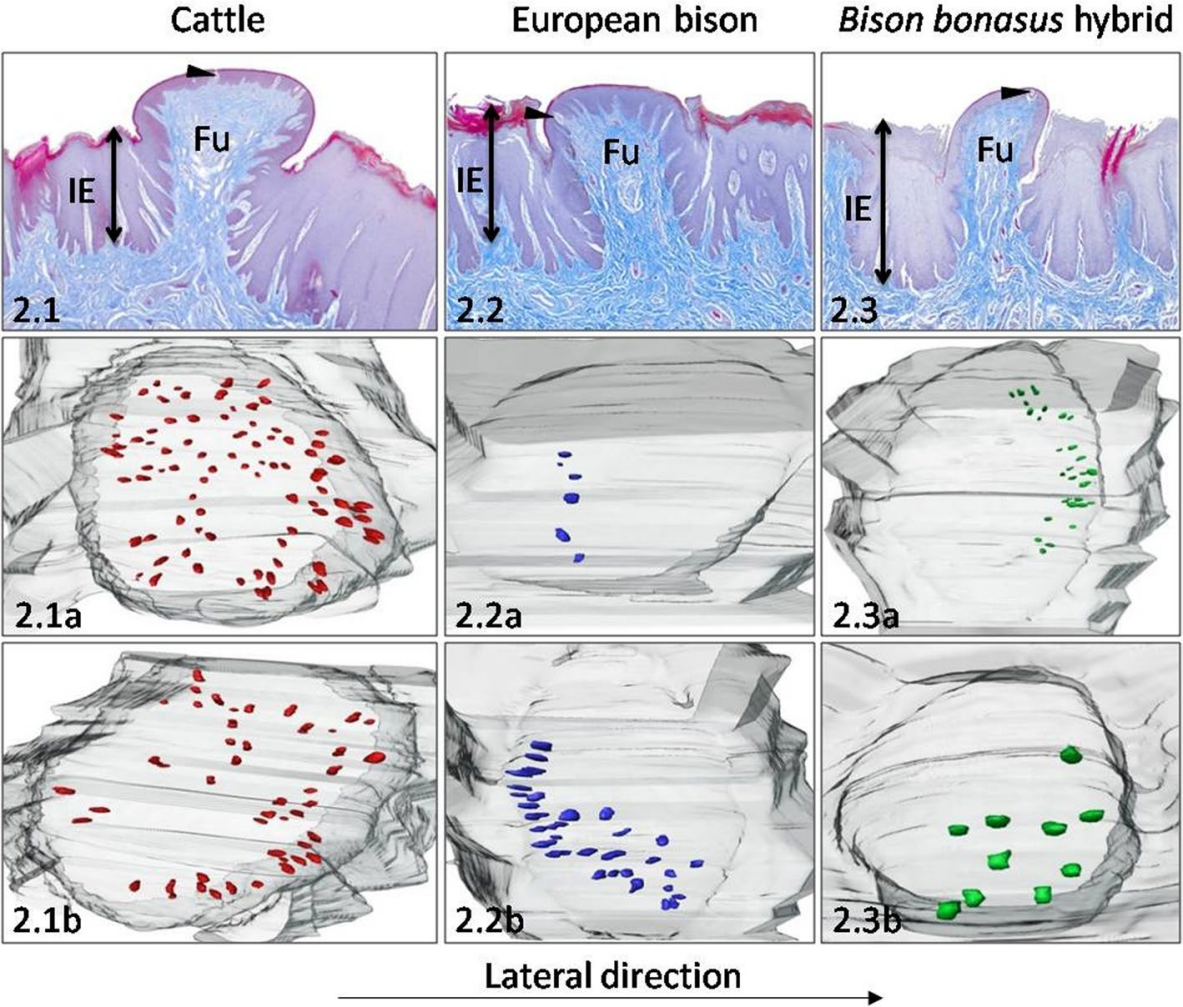
Ventral surface of the apex. The Fu papillae and its three-dimensional reconstructions on the ventral surface of the apex in cattle (**2.1**), European bison (**2.2**), and *Bison bonasus* hybrid (**2.3**). Fu – fungiform papillae, IE – interpapillary epithelium, black arrowhead – taste buds. Taste buds on 3D models – red dots (cattle), blue dots (European bison), green dots (*Bison bonasus* hybrid)

In European bison, 50 Fu papillae covered both left and right marginal areas of the ventral surface of the tongue (Table 1). The density of these elliptical or rounded papillae was 2.6 papillae/cm^2^ (Table 1, Fig. 2.2). Generally, Fu papillae had flat, smooth dorsal surface, and the diameter ranged between 552 μm and 1212 μm (Table 2, Fig. 2.2). The difference between the height of Fu papilla and the interpapillary epithelium equals 245.6 μm, which was the mean value of papillary protrusion (Tables 2 and 4, Fig. 8A). Obtained diameter of 3D models of CTC revealed its columnar-like shape (Table 3, Figs. 2.2, 7B and 8B). The stratified squamous epithelium was on average 139.5 μm high, while its keratinised layer accounted 10.6% of the total epithelium thickness (Table 4, Fig. 8C). The taste buds in European bison were arranged anteriorly and near the right border of the dorsal surface of Fu papillae (Fig. 2.2a, 2.2b). Their average number on two 3D models was 18 taste buds, while their estimated density reached 20 taste buds/mm^2^ (Table 1). The average diameter of taste buds was 41.7 μm (Table 5).

A total of 220 Fu papillae were arranged in a V-letter on the *Bison bonasus* hybrid tongue (Table 1). Additionally, two types of Fu papillae were observed. Bigger Fu papillae created a border between smooth area and area covered by lingual papillae, while smaller Fu papillae were spread between filiform papillae. Its density reached 20 Fu papillae/cm^2^ (Table 1). Fu papillae were elliptical or round in outline with a mean diameter of 664.9 μm (Table 2, Fig. 2.3). Its dorsal surface was convex and smooth (Fig. 2.3). The height of Fu papillae, interpapillary stratified epithelium, and the papillary protrusion reached 1104 μm, 930.1 μm, and 173.9 μm, respectively (Tables 2 and 4, Fig. 8A). There were observed columnar-like shape CTCs (Table 3, Figs. 2.1, 7B and 8B). The thickness of the epithelium on Fu papillae was 96.9 μm, of which 9.5% was the keratinized layer (Table 4, Fig. 8C). The patterns of 20 taste buds in average in *Bison bonasus* hybrid were arranged posteriorly in a crescent shape and on the right part of the dorsal surface of Fu papillae (Table 1, Figs. 2.3a, 2.3b). The estimated density of taste buds was 21 taste buds/ mm^2^ (Table 1). In turn, their mean diameter reached 42.6 μm (Table 5).

### Dorsal surface of the lingual body

In cattle and European bison, Fu papillae were observed on the lateral areas of the lingual body. In contrast, in the *Bison bonasus* hybrid Fu papillae were distributed on the whole dorsal surface of the lingual body.

In cattle observed 35 Fu papillae with the density of 0.8 papilla/cm^2^ (Table 1). Round or elliptical Fu papillae in outline with convex and smooth dorsal surface was 1173.1 μm in diameter (Table 2, Fig. 3.1). The Fu papillae height was 1343.9 μm, while the interpapillary epithelium – 1096.9 μm, so the papillary protrusion equals 247 μm (Tables 2, 4, Fig. 8A). The diameters of CTC characterized the balloon-like shape visible in Fig. 7A (Table 3, Figs. 3.1 amd 8B). The thickness of the epithelium on Fu papillae in this area of tongue was 122.3 μm with the keratinized layer, which made up 13.2% of the entire epithelium thickness. (Table 4, Fig. 8C). The taste buds observed on 3D models of Fu papillae in cattle formed perpendicular or parallel strips concerning the median line of the tongue (Fig. 3.1a, 3.1b, 3.1c). Averaged number of taste buds was 41, while its estimated density reached 22 taste buds/mm^2^ (Table 1). In turn, the measured diameter of taste buds ranged between 30.2 μm – 55.6 μm (Table 5).

**Fig. 3:**
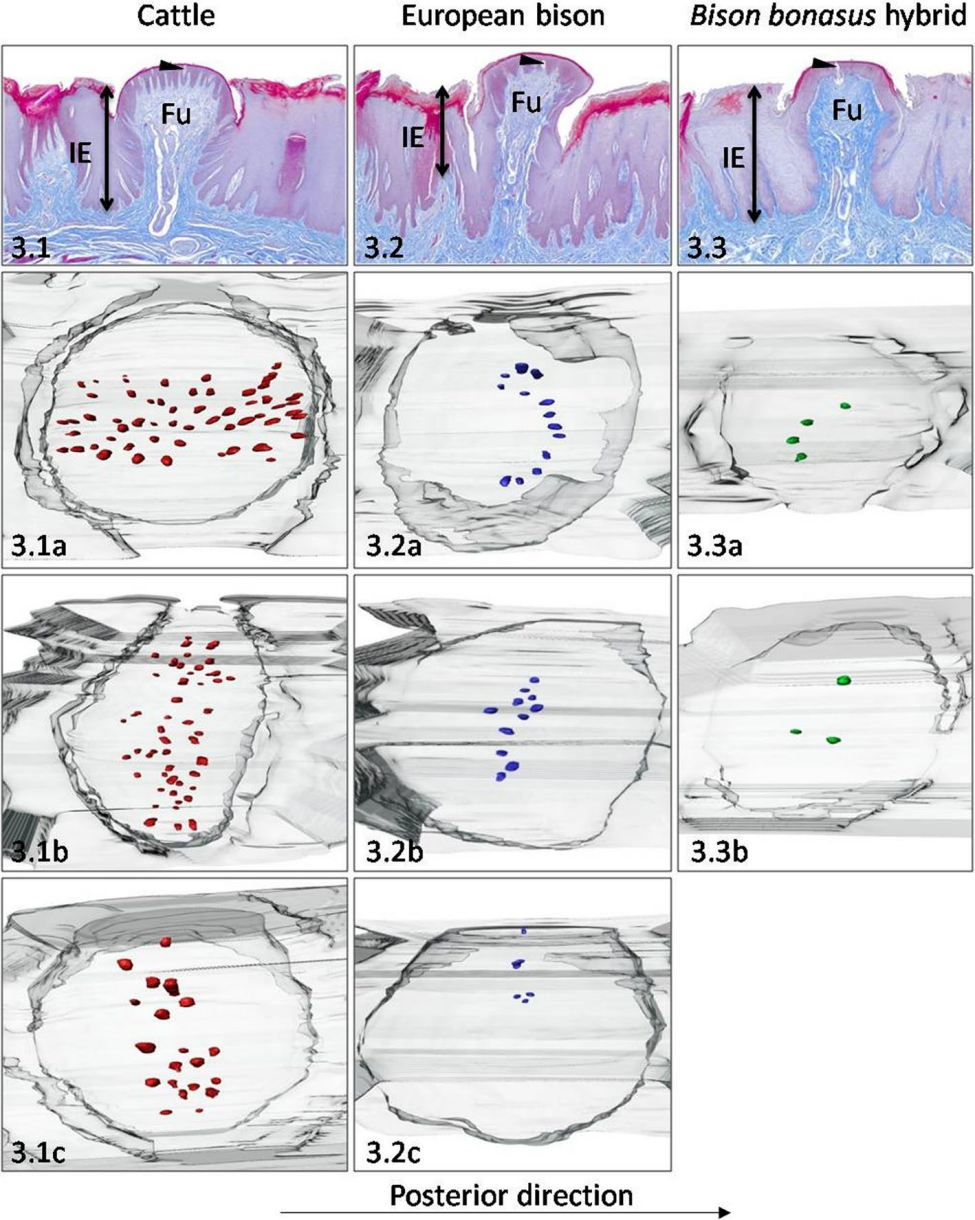
Dorsal surface of the lingual body. The Fu papillae and its three-dimensional reconstructions on the dorsal surface of the lingual body in cattle (**3.1**), European bison (**3.2**), and *Bison bonasus* hybrid (**3.3**). Fu – fungiform papillae, IE – interpapillary epithelium, black arrowhead – taste buds. Taste buds on 3D models – red dots (cattle), blue dots (European bison), green dots (*Bison bonasus* hybrid)

In European bison, the number and density of Fu papillae were 32 and 1 papilla/cm^2^, respectively (Table 1). The Fu papillae with a mean diameter of 1102.5 μm were round in outline with a convex and smooth dorsal surface (Table 2, Fig. 3.2). The average height of these papillae and the interpapillary epithelium reached 1508.2, and 1067.1 μm, respectively (Tables 2, 4). Thus, the protrusion of Fu papillae was 441.1 μm (Fig. 8A). In regard to obtained dimensions, the CTC was columnar-like in shape (Table 3, Figs. 3.2, 7B and 8B). The thickness of the epithelium on Fu papillae was 166.5 μm, while the keratinized layer was 12% of its thickness (Table 4, Fig. 8C). In European bison, the arrangement of taste buds on the dorsal surface of the Fu papillae was different in each of the reconstructed papillae. On the first one, taste buds were arranged posteriorly in a crescent shape (Fig. 3.2a). On the second one, taste buds formed a centrally located strip arranged perpendicularly to the median line of the tongue (Fig. 3.2b). In turn, on the third one, a group of taste buds was located on the left part of the dorsal surface of Fu papilla (Fig. 3.2c). The average number of taste buds from these three models equals 10, as same as the estimated density - 10 taste buds/mm^2^ (Table 1). Moreover, the measured diameter of taste buds was 42.4 μm (Table 5).

In the *Bison bonasus* hybrid the number and density of Fu papillae reached 118 and 3 Fu papillae/cm^2^, respectively (Table 1). These papillae were round in outline with flat and smooth dorsal surface with the mean diameter of 828.9 μm (Table 2, Fig. 3.3). Its average height ranged between 1269 μm and 1380 μm (Table 2). Thus, analysing the thickness of the interpapillary epithelium, the protrusion of Fu papillae with the value of 101.2 μm were estimated (Table 4, Fig. 8A). The Fu papillae CTC on the hybrid’s lingual body was characterized by a balloon-like shape (Table 3, Figs. 3.3, 7A and 8B). The mean value of the thickness of the epithelium covering Fu papillae was 118.6 μm, while the keratinized layer represented 16% of its thickness and was higher as in parental species (Table 4, Fig. 8C). In the *Bison bonasus* hybrid, two 3D models of Fu papillae had centrally arranged taste buds (Fig. 3.3a, 3.3b). The mean number of taste buds reached 3, while their density was five taste buds/mm^2^ of the papilla surface (Table 1). Measured and averaged diameter of taste buds was 45.2 μm (Table 5).

### Dorsal surface of the medial part of the torus

On the torus in cattle, European bison and *Bison bonasus* hybrid Fu papillae were observed on the medial and posterior part of the dorsal surface and on its lateral surfaces.

In cattle on the dorsal surface of the torus observed 15 Fu papillae with a density of 0.5 papilla/cm^2^ (Table 1). In outline, Fu papillae were elliptical with 1865.6 μm in diameter, whereas their surface was flat and undulated (Table 2, Fig. 4.1). The height of Fu papillae, the interpapillary stratified epithelium, and papillary protrusion reached 1319.8 μm, 1006.9 μm, and 312.9 μm, respectively (Tables 2 and 4, Fig. 8A). The diameters of CTC characterized the cone-like shape, visible on Fig. 7C (Table 3, Figs. 4.1 and 8B). In addition, Fu papillae were covered by 143.2 μm of stratified squamous epithelium, in which the keratinized layer was 20% of its thickness (Table 4, Fig. 8C). On 3D models of Fu papillae in cattle 13 taste buds formed clusters arranged posteriorly or centrally on the dorsal surface of Fu papillae (Table 1, Fig. 4.1a, 4.1b). The density of taste buds reached five taste buds/mm^2^, while the diameter was 41.1 μm (Tables 1 and 5).

**Fig. 4:**
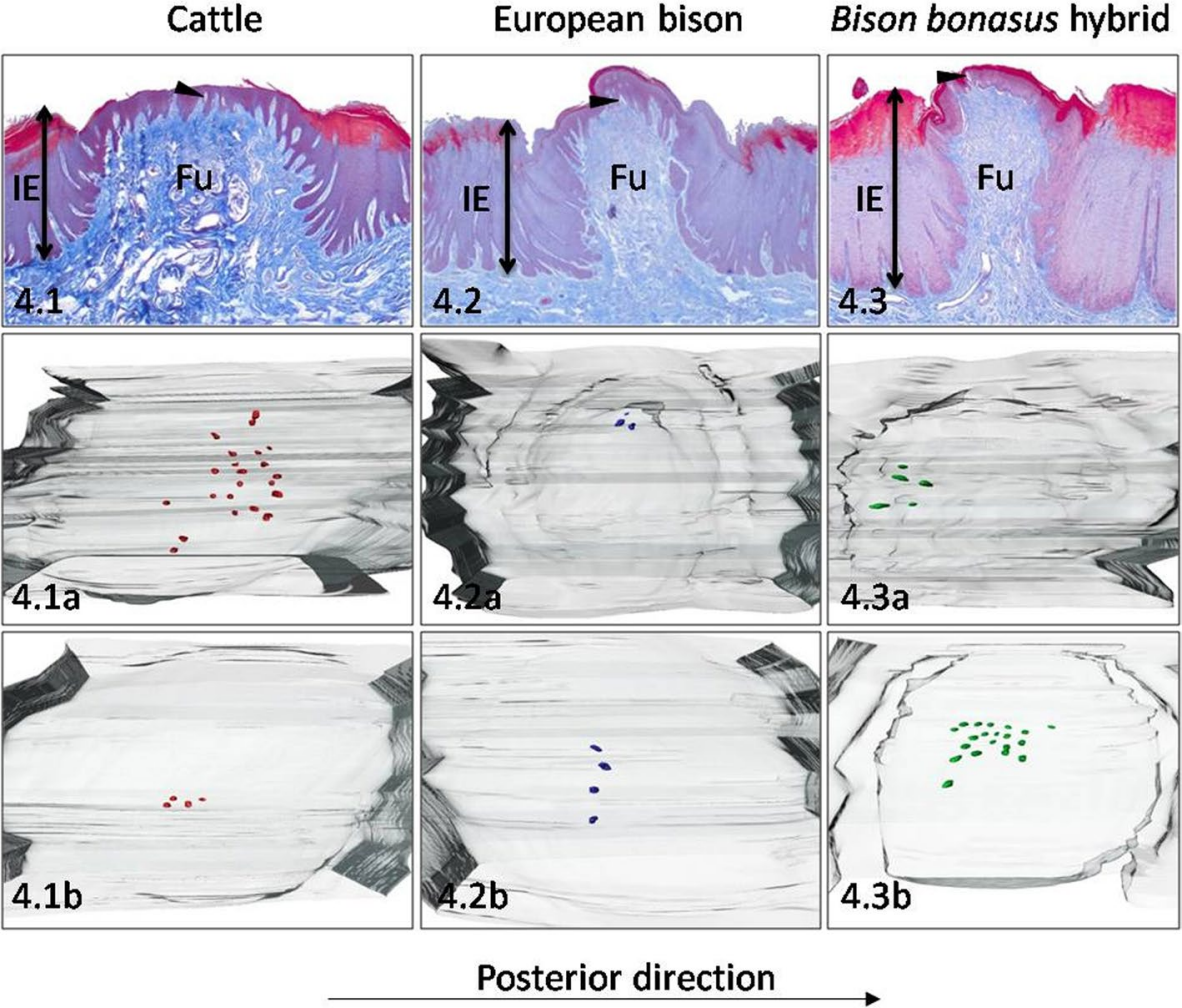
Dorsal surface of the medial part of the torus. The Fu papillae and its three-dimensional reconstructions on the dorsal surface of the medial part of the torus in cattle (**4.1**), European bison (**4.2**), and *Bison bonasus* hybrid (**4.3**). Fu – fungiform papillae, IE – interpapillary epithelium, black arrowhead – taste buds. Taste buds on 3D models – red dots (cattle), blue dots (European bison), green dots (*Bison bonasus* hybrid)

In European bison, the number and density of pigmented Fu papillae were 23 and 0.8 papilla/cm^2^, respectively (Table 1). The dorsal surface of the elliptical in outline Fu papillae was convex and irregular, while their diameter reached 1628 μm (Table 2, Fig. 4.2). The comparison of the height of Fu papillae and interpapillary epithelium indicated the value of the papillary protrusion, which equals 327 μm (Tables 2 and 4, Fig. 8A). In turn, measured diameters of CTC in the dorsal, medial and basal parts presented its cone-like shape (Table 3, Figs. 4.2, 7C and 8B). The thickness of stratified squamous epithelium on Fu papillae was 150.6 μm, while the keratinized layer represented 24.6% of the epithelium thickness (Table 4, Fig. 8C). In European bison only four taste buds were arranged on the left or central part of the dorsal surface of Fu papillae (Table 1, Fig. 4.2a, 4.2b). The estimated density of taste buds was two taste buds/mm^2^, whereas its mean diameter equals 45.1 μm (Tables 1 and 5).

The number and density of Fu papillae on the dorsal surface of the torus in the *Bison bonasus* hybrid reached 15 and 0.6 papilla/cm^2^, respectively (Table 1). Fu papillae were round in outline with flat and smooth surface and had 1357.7 μm in diameter (Table 2, Fig. 4.3). The Fu papillae were 2060.1 μm in height, while the height on the interpapillary stratified epithelium was 1897.8 μm (Tables 2 and 4). Thus, the protrusion of the papillae equals 162.3 μm (Fig. 8A). The CTC with a wide dorsal part had a balloon-like shape (Table 3, Figs. 4.3, 7A and 8B). The thickness of stratified squamous epithelium on the Fu papillae was 121.9 μm, of which 29% represented the keratinized layer, and at the same time it was the highest value among studied ruminants (Table 4, Fig. 8C). In *Bison bonasus* hybrid on two 3D models of Fu papillae, 13 taste buds arranged anteriorly were observed (Table 1, Fig. 4.3a, 4.3b). The mean density of taste buds was 11 taste buds/mm^2^, whereas its diameter was 42.2 μm in average (Tables 1 and 5).

### Dorsal surface of the posterior part of the torus

On the posterior part of cattle’s torus observed 30 Fu papillae, with a density of 0.9 papillae/cm^2^ (Table 1). Fu papillae were rounded in outline, with a convex dorsal surface withan average diameter of 1095.7 μm (Table 2, Fig. 5.1). Analysing the height of Fu papillae and the interpapillary epithelium, the papillary protrusion of 932.1 μm was estimated (Tables 2 and 4, Fig. 8A). The CTC were the balloon-like shape (Table 3, Figs. 5.1, 7A and 8B). The average thickness of the stratified epithelium on the Fu papillae ranged between 47 μm – 193 μm, while the keratinized layer was 15% of the epithelium thickness (Table 4, Fig. 8C). The average number of 37 taste buds evenly spread on the dorsal surface of the Fu papillae in cattle present in Table 1, and Fig. 5.1a, 5.1b. Its estimated density reached 24 taste buds/mm^2^, while its mean diameter ranged between 30.1 μm – 49.4 μm (Tables 1, 5).

**Fig. 5:**
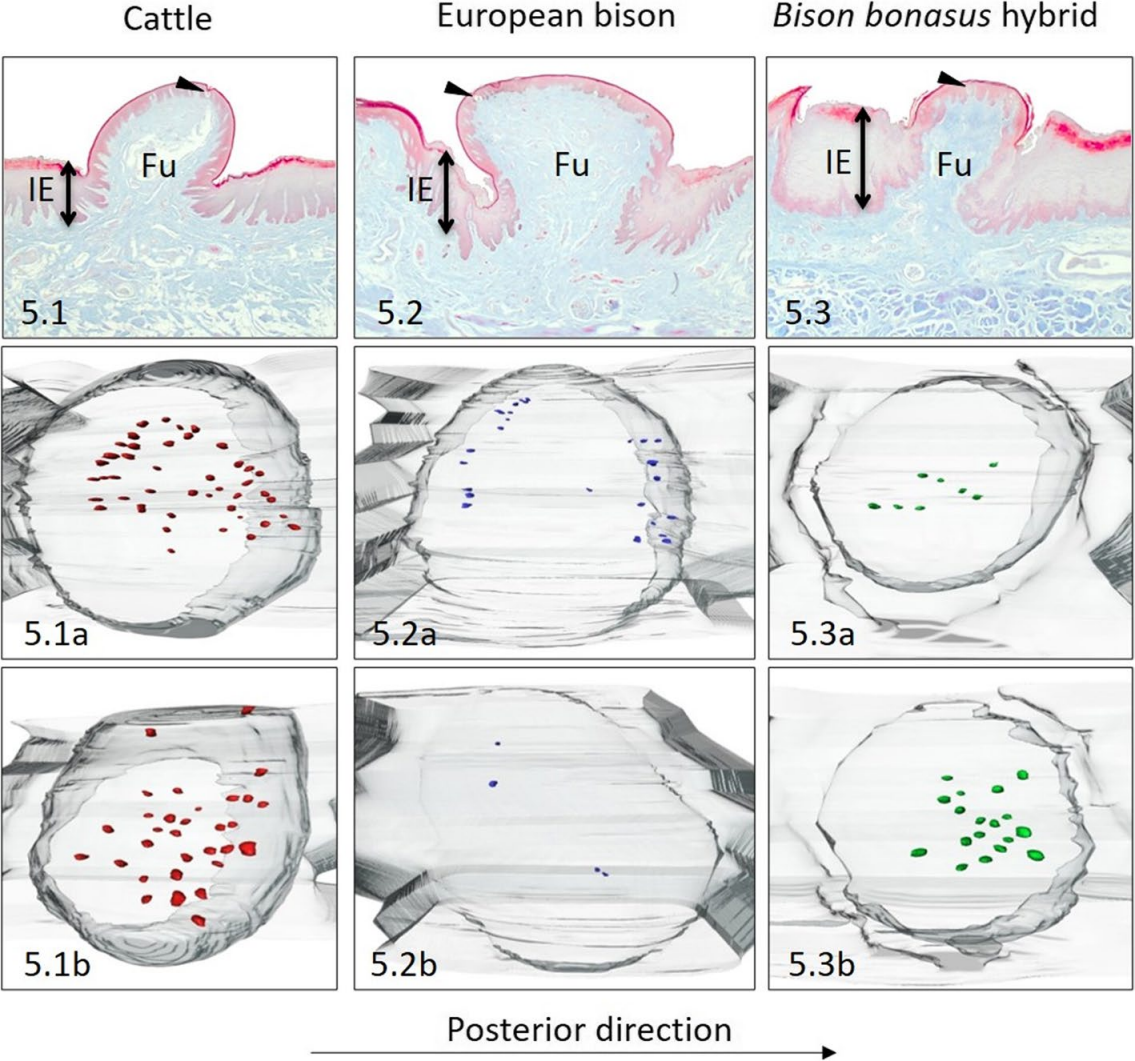
Dorsal surface of the posterior part of the torus. The Fu papillae and its three-dimensional reconstructions on the dorsal surface of the posterior part of the torus in cattle (**5.1**), European bison (**5.2**), and *Bison bonasus* hybrid (**5.3**). Fu – fungiform papillae, IE – interpapillary epithelium, black arrowhead – taste buds. Taste buds on 3D models – red dots (cattle), blue dots (European bison), green dots (*Bison bonasus* hybrid)

Like cattle, the estimated density of Fu papillae in European bison was 0.9 papilla/cm^2^, but the number of these papillae was smaller as in cattle and reached 20 (Table 1). The dorsal part of rounded or elliptical in outline Fu papillae was also convex and smooth, while the diameter ranged between 1134 μm - 1883 μm (Table 2, Fig. 5.2). The papillary protrusion obtained from the difference in the height of studied papilla and interpapillary epithelium equals 706.7 μm (Tables 2 and 4, Fig. 8A). The columnar-like shape of CTC was characterized by similar diameters of this structure in its basal, medial and dorsal parts (Table 3, Figs. 5.2, 7B and 8B). The stratified squamous epithelium was 129.5 μm in height, while the keratinized layer with the value of 19.2 μm represented 14.8% of its thickness (Table 4, Fig. 8C). In European bison in average 14 taste buds were spread unevenly on the anterior, posterior, and lateral parts of the Fu papillae, creating small clusters (Table 1, Fig. 5.2a, 5.2b). The density of taste buds was four taste buds/mm^2^, while its mean diameter reached up to 42.9 μm (Tables 1 and 5).

The number and density of rounded and convex Fu papillae in the *Bison bonasus* hybrid was 46 and 2.7 papillae/cm^2^, respectively (Table 1, Fig. 5.3). The diameter of Fu papillae amounted to 895 μm (Table 2). The height of studied papillae reached 1221.5 μm, while the height of interpapillary stratified epithelium was 944.4 μm, so the papillary protrusion equals 277.1 μm (Tables 2 and 4, Fig. 8A). In turn, CTC had a balloon-like shape concerning its diameters in basal, medial and dorsal part (Table 3, Figs. 5.3, 7A and 8B). Furthermore, Fu papillae were covered with stratified squamous epithelium 113.3 μm high, in which the keratinized layer represented 14.8% of his thickness (Table 4, Fig. 8C). Around 13 taste buds formed vertical and circular clusters on the dorsal surface of Fu papillae in the *Bison bonasus* hybrid (Table 1, Fig. 5.3a, 5.3b). The estimated density of these taste buds was 11 taste buds/mm^2^, whereas its diameter was 36.5 μm (Tables 1 and 5).

### Lateral surfaces of the torus

The 45 Fu papillae with a density of 0.9 papilla/cm^2^ were regularly distributed on the lateral surfaces of cattle torus (Table 1). The diameter of round Fu papillae with a convex and smooth surface reached 1071.7 μm (Table 2, Fig. 6.1). The differences in the height of the papillae and interpapillary stratified epithelium indicated the papillary protrusion to a height of 108.7 μm (Tables 2, 4, Fig. 8A). The 3D model of CTC revealed cone-like shape in studied papillae Fu papillae (Table 3, Figs. 6.1, 7D and 8B). The thickness of stratified squamous epithelium on Fu papillae amounted to 147.1 μm, whereas the thickness of his keratinized layer was expressed by 21.4% of his thickness, what represents the highest value for cattle (Table 4, Fig. 8C). In cattle, 17 taste buds were arranged centrally and on the left side of the dorsal surface of Fu papillae (Table 1, Fig. 6.1a, 6.1b). The density of taste buds in Fu papillae reached 16 taste buds/mm^2^ in average (Table 1). Furthermore, the mean diameter of taste buds amounted to 44.8 μm (Table 5).

**Fig. 6:**
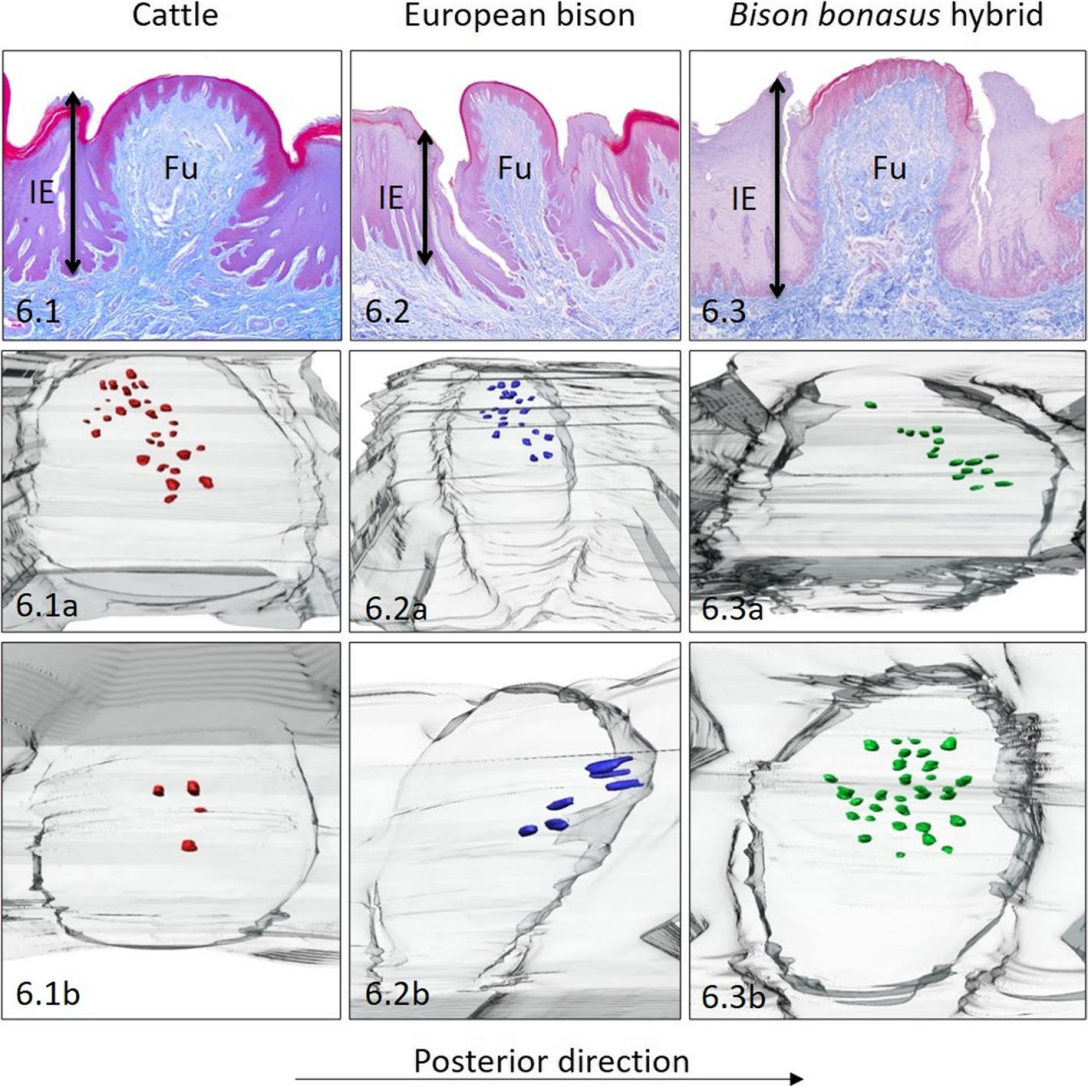
Lateral surfaces of the torus. The Fu papillae and its three dimensional reconstructions on lateral surfaces of the torus in cattle (**6.1**), European bison (**6.2**) and *Bison bonasus* hybrid (**6.3**). Fu – fungiform papillae, IE – interpapillary epithelium, black arrowhead – taste buds. Taste buds on 3D models – red dots (cattle), blue dots (European bison), green dots (*Bison bonasus* hybrid)

**Fig. 7:**
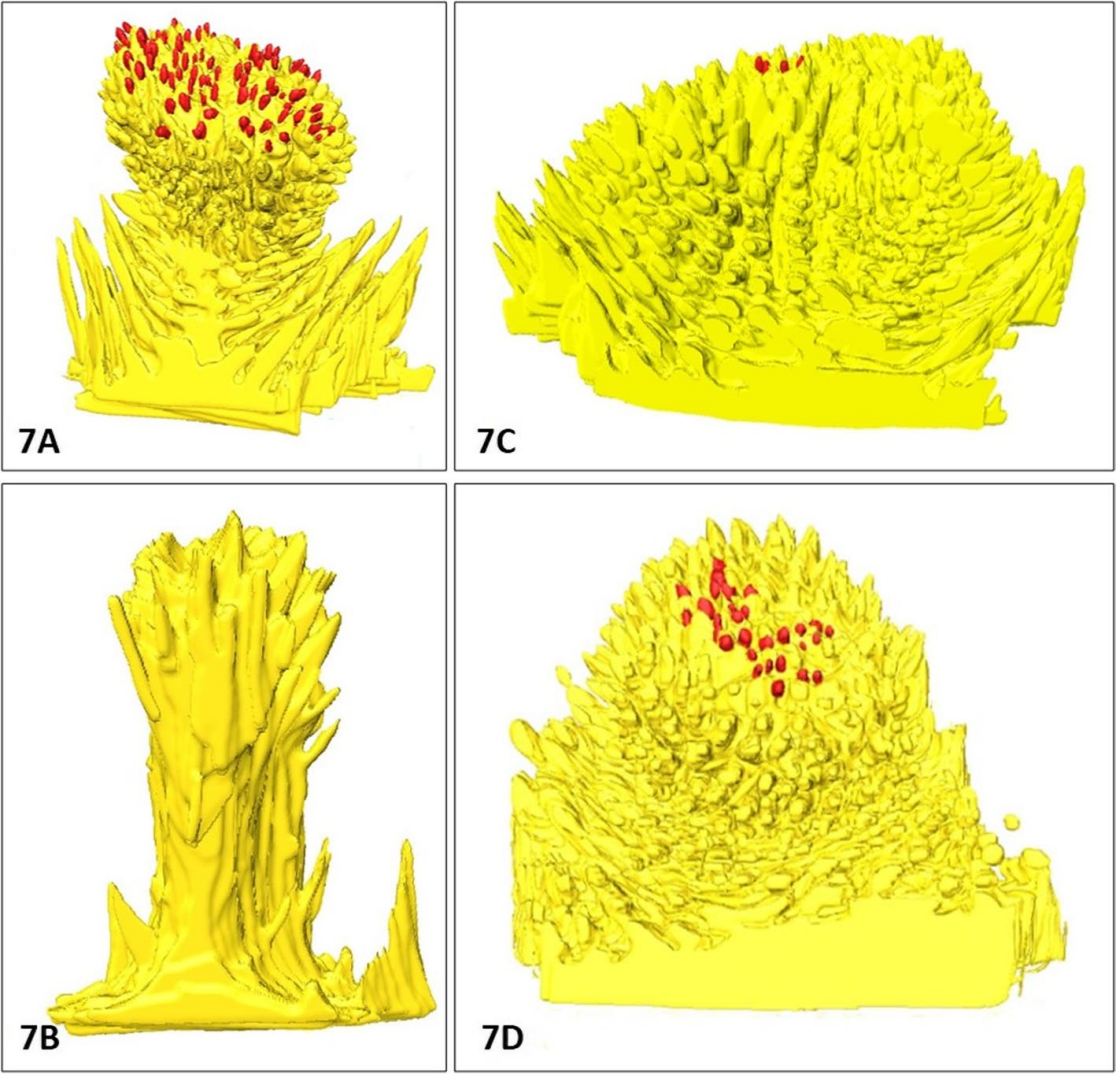
The types of connective tissue cores (CTC) of Fu papillae in cattle, European bison, and *Bison bonasus* hybrid. **7A** – balloon-like CTC on the ventral surface of the apex in cattle, **7B** - columnar-like CTC on the dorsal surface of the apex in *Bison bonasus* hybrid, **7C** - cone-like CTC on the dorsal surface of the torus in cattle, **7D** - cone-like CTC on lateral surfaces of the torus in cattle. Red dots – taste buds

**Fig. 8:**
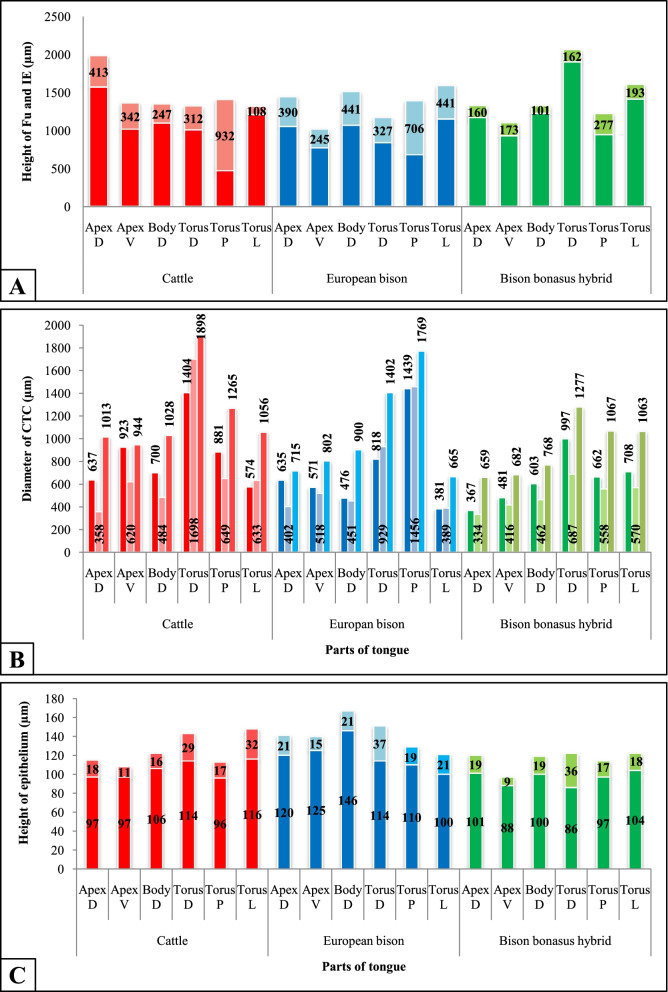
Graphical results of calculated data obtained during conducted studies in cattle, European bison, and *Bison bonasus* hybrid. **A** the height of Fu papillae and interpapillary epithelium (IE) with the papillary protrusion (brighter colors) above the interpapillary epithelium (darker colors), **B** the diameter of connective tissue cores (CTCs) on its dorsal (the darkest colors), medial (the brightest colors), and basal (intermediate colors) part in Fu papillae, **C** the height of stratified squamous epithelium and its keratinized layer (brighter colors) on the apical surface of Fu papillae. Apex D – dorsal surface of the apex, Apex V – ventral surface of the apex, Body D – dorsal surface of the lingual body, Torus D – dorsal surface of medial part of torus, Torus P – dorsal surface of posterior part of torus, Torus L – lateral surfaces of torus

In European bison number of Fu papillae on the left and right surface of torus was 46, while the density reached 1.1 papilla/cm^2^ (Table 1). The dorsal part of the elliptical in outline Fu papillae was convex and smooth, while their diameter equalled 655.6 μm (Table 2, Fig. 6.2). The average heights of Fu papillae and interpapillary stratified epithelium were 1589 μm, and 1147.8 μm, respectively (Tables 2 and 4). Thus, the protrusion of Fu papillae over the epithelium was 441.2 μm (Fig. 8A). The columnar-like shape of CTC was observed (Table 3, Figs. 6.2, 7B and 8B). The epithelium covering Fu papillae was 1020.9 μm in high, of which 17% represented keratinized layer (Table 4, Fig. 8C). On two 3D reconstructions of Fu papillae in European bison clusters of 14 in average taste buds were observed on the left side and posterior part of surface of Fu papilla (Table 1, Fig. 6.2a, 6.2b). The density of these taste buds is 18 taste buds/mm^2^ (Table 1). Additionally, measured diameter of taste buds ranged between 23.8 μm – 56.1 μm (Table 5).

The total number of Fu papillae in *Bison bonasus* hybrid was 38, while the density reached 1.06 papilla/cm^2^ (Table 1). Round or elliptical in outline Fu papillae with flat and smooth dorsal surface were 964 μm in diameter and 1601.9 μm in height (Table 2, Fig. 6.3). The height of the interpapillary epithelium was 1408.9 μm, so the papillary protrusion was 193 μm (Table 4, Fig. 8A). The wider dorsal diameter of Fu papilla CTC determined the balloon-like shape of this structure (Table 3, Figs. 6.3, 7A and 8B). Fu papillae were covered by stratified squamous epithelium 121.2 μm in high, of which the 14.6% represented his keratinized layer (Table 4, Fig. 8C). In the *Bison bonasus* hybrid, taste buds were arranged posteriorly or centrally, close to the left area of the dorsal surface of Fu papillae (Fig. 6.3a, 6.3b). The 23 taste buds were observed in average, while its density reached 19 taste buds/mm^2^ (Table 1). Moreover, the mean diameter of those taste buds was 36.9 μm (Table 5).

## Discussion

The present microstructural study of the Fu papillae and its taste buds in cattle, European bison, and *Bison bonasus* hybrid deepens the data about the diversity of gustatory papillae revealed during earlier morphological analyses about tongues in mentioned species [27]. Based on previous data of Fu papillae on tongues between parental species and hybrid, the amount and density of Fu papillae were estimated. In cattle and the European bison the total number of the papillae over the tongue was comparable, whereas the amount Fu papillae in the *Bison bonasus* hybrid was ca 3.4 times higher than its parental species. So far, the amount of Fu papillae among mammals was estimated in some species of insectivores, rodents, carnivores, ruminants, and humans. The number of Fu papillae was 10-12 in the common shrew, 150 - 300 in mice, 114 - 221 in rat, 500 in opossum, 240 - 260 in cat, 250 in the dog, 424 - 442 in sheep, 200-250 in bovine, and around 200 in human [13, 43–47].

The obtained data showed that in parental species, 51 to 59% of the Fu papillae were situated on the anterior part of the tongue, i.e., apex and body, while in the *Bison bonasus* hybrid, this value goes up to 85%. Additionally, the number of Fu papillae vary between the dorsal and ventral surface of the lingual apex. In parental species, the amount of Fu papillae on the ventral surface of the tongue is higher concerning the dorsal surface. However, the number of Fu papillae in the *Bison bonasus* hybrid on the dorsal and ventral surface of the lingual apex was almost equal and was on average 86 and 82% higher than in parental species, i.e., cattle, and European bison, respectively. Moreover, the estimated density of Fu papillae on the ventral surface of the apex in *Bison bonasus* hybrid reached up to 50 times more concerning other studied parts of the tongue of cattle and European bison. On the other hand, the uneven distribution of Fu papillae on the lingual apex was earlier observed in other studied ruminants i.e., alpaca, goat, Iraqi goat, fallow deer, and Egyptian buffalo [20, 23, 33, 48, 49].

According to behavioral studies, the relation of the mechanism of feeding and content of consumed food in ruminants, it can be assumed that the ventral surface of the apex with an accumulation of Fu papillae in studied ruminants is the particular contacting area of food preselection before taking a bite [23, 50]. The second relevant part of the tongue in ruminants is the lingual torus due to the mastication of food. During cyclic side-by-side jaw movements, fine particles regurgitated from rumen are again ground by the teeth and rubbed between the surface of the tongue and the palate. During this displacement of food over the torus surface, the sensory system of Fu papillae is activated. For the first time, our analyses characterized Fu papillae on the dorsal and lateral surfaces of the torus of the tongue with particular emphasis on the posterior dorsal part of the torus. We revealed that the number and density of papillae increase from the medial to the posterior area on the dorsal surface of the torus in cattle and *Bison bonasus* hybrid by approximately 50-67%. In contrast, in European bison, these values were almost constant.

Interesting results observed in parental species were that the number and density of Fu papillae on lateral surfaces of the torus of the tongue excessed values noted on the posterior part of the torus. Thus, statements about the posterolateral surfaces of the lingual torus identify them as important food tasting areas during long chewing cycles, because of Fu papillae and numerous Vp papillae. It gives essential advice for further studies in ruminants to take samples not only from the medial dorsal surface of the torus, which is often considered as representative area, but also from posterior and lateral surfaces of the torus.

The Fu papillae observed on 3D models in cattle, European bison, and *Bison bonasus* hybrid were predominantly round in outline with a convex or flattened dorsal surface. In mammalian species such as yak, cattle, cattle-yak, Iraqi goat, Iranian buffalo, hippopotamus, koala, arctic fox, Japanese badgers, and wild boar, the Fu papillae were described as mushroom- or dome-shaped [3, 8, 9, 17, 18, 21, 25, 31, 33]. The diameters of Fu papillae in studied ruminants vary over the tongue. However, the diameters of Fu papillae in the anterior part of the tongue were lower than on torus, from 18% in cattle, 23% in European bison to even 32% in *Bison bonasus* hybrid.

Comparing the height of Fu papillae in cattle, European bison, and their hybrid, we stated that only in cattle, the Fu papillae in the anterior part of the tongue was about 16% higher than on the torus. In contrast, in European bison and hybrid, the height of Fu papillae on the torus was higher by 5 and 23%, respectively. The estimating protrusion of the dorsal part of Fu papillae over the interpapillary stratified epithelium may be a good index explaining the rate of exposure of the dorsal surface of Fu papillae, containing taste buds, to taste stimuli due to food selection and/or control of swallowed food. In the studied species, the average protrusion of papillae was 392 μm in cattle, 425 μm in European bison, and only 177 μm in European bison hybrid. On the anterior dorsal part of the tongue were note lower values of papillary protrusion compared to torus by approximately 46% in cattle, 20% in European bison, and 41% in *Bison bonasus* hybrid. In parental species, the papillary protrusion declined between the dorsal and ventral surface of the apex by 17% in cattle and 37% in European bison. In contrast, in the *Bison bonasus* hybrid, this protrusion increase by ca. 8% than the dorsal surface.

The correlated observations of 3D models parallel with serial 2D histoslides gave an occasion to the comparative description of two elements of Fu papillae, i.e., superficial epithelium, which contains taste buds and connective tissue cores (CTC).

The keratinized stratified squamous epithelium protects Fu papillae in ruminants against mechanical damage during chewing and rumination of food. According to our analyses, the average height of stratified squamous epithelium on Fu papillae was the highest in European bison, which may relate to the access to structurally diverse food available in a wild environment, changing with seasons. It is opposite to livestock, which feeds on structurally similar forage. The highest papillary epithelium coverFu papillae on the medial part of the torus in European bison and its hybrid, and the lowest values were noted on the ventral surface of the apex. The average thickness of a keratinized layer of epithelium on Fu papillae in the studied species is 21 μm, which is ca. 16% of the total height of the epithelium. In contrast, on the ventral surface of the tongue, these values decrease to 9-14 μm and 9.5-10.5% what is important in exposes taste pores on the Fu papillae surface during the food selection.

The connective tissue core (CTC) of Fu papillae are the internal skeleton for capillary networks and nerve bundles, strongly attached with mucosal epithelium. So far, the shapes of CTCs as collagenous skeleton were studied in scanning electron microscopy after NaOH maceration of epithelia and free cells of connective tissues in rabbit, koala, hippopotamus, goat, cattle, Reeves’ muntjac deer, Japanese badgers, pig, or primates [3, 4, 7, 9, 25, 28, 51]. These results revealed various shapes of CTCs, named flower-like, balloon-like, cauliflower-like, pine-cone-like, or columnar-like [3, 4, 7, 9, 25, 28, 51, 52].

Correlative morphometric analysis and 3D modeling of Fu papillae allowed to distinguished rounded in outline Fu papillae with three types of CTCs. The balloon-shaped CTCs were characterized by wide base narrowing in the medial part and often expanding near or over the surface of the interpapillary epithelium. Columnar-like type of CTC have quite similar dimensions on its dorsal and medial part, but some wider on the basal part, whereas in cone-like CTC, the width becomes smaller from base to the top of papillae. There were all three CTC types in European bison, whereas in cattle, only balloon-like and cone-like CTCs. The columnar and balloon-like CTCs were typical for *Bison bonasus* hybrid.

Analyzing the spatial distribution of Fu papillae and their CTC we revealed that on both surfaces of the lingual apex in each examined ruminant are balloon-like or columnar-like CTCs. On the torus of the tongue in cattle and European bison, there were often the cone-like CTCs, which correspond with smaller protrusion of Fu papillae over the mucosa surface. However, in European bison hybrid on all surfaces of lingual torus were only balloon-like types of CTCs. On the surface of CTC models in all studied species, separate elongated connective tissue processes, get deep into the thick interpapillary epithelium. Such superficial microstructures in shape/form of vertical plices, folds, and striations providing the well attachment of the epithelia were studied previously in scanning electron microscopy [3, 4, 7, 9, 25, 28, 51]. According to Yoshimura et al. (2009) there was a primary core with numerous secondary cores in CTC of Fu papillae. Moreover, around the CTCs of Fu papillae, many separate papillary cores of the interpapillary epithelium were observed [25]. The observations of 3D CTCs models in studied ruminants showed on the surface of primary cores of a balloon- and cone-like CTC numerous short secondary cores, whereas these connective tissue structures in columnar-like CTC are rare and slender. In addition, the separate elongated intrapapillary cores were observed in the basal part of the balloon- and columnar-like type of CTCs.

During the microstructural studies of Fu papillae in mammalian species, in determining the number and distribution of taste buds in these papillae, three methods so far were used. The analyses of taste buds were primarily based on 2D observations of the cross-sections of Fu papillae or counting of taste pores visible on scanning electron microscopic electronograms. Such studies were conducted in Egyptian fruit bats, wild boar, pig, hippopotamus, goat, yak, wolf, and Japanese badgers [9, 15, 18, 25, 31, 34, 49]. The third method was indirect because of the NaOH macerated CTC of Fu papillae. The depressions on the dorsal surface of CTC were considered as places for taste buds [3, 7].

In current studies, we applied for the first time the three-dimensional analysis of taste buds allowed on a spatial description of distribution and counting of taste buds as a more efficient method used to describe the taste buds system in Fu papillae. Observations of serial histoslides resulted in determining the diameter of taste buds, ranging from 33 μm to 48 μm, which may specify the size of taste buds for ruminants independent of the thickness of the stratified squamous epithelium. Studies on 3D models of Fu papillae with marked taste buds revealed that among three examined ruminants number of taste buds differs from 1 up to 64. On the dorsal surface of the apex in the *Bison bonasus* hybrid, were found no taste buds in Fu papillae.

Previously, the number of taste buds counted from histoslides in mammalian species was often described as “numerous, several or few” taste buds [4, 9, 18, 21, 33, 41]. Estimating the number of Fu papillae and taste buds per single papilla on 3D models enabled us to calculate an approximate number of taste buds on the whole tongue. The number of taste buds was the highest in cattle, and in *Bison bonasus* hybrid and equals 6659 and 6620, respectively, while in European bison was estimated ca. 60% fewer taste buds, i.e., 2608. Previous publication about the number of taste buds in bovine was estimated from 1580 to 1838 taste buds in Fu papillae, which seems underestimated compared to our results [43]. In other mammalian species, like Akkaraman sheep, described 1165-1243 taste buds, while in puppy 1444 taste buds in Fu papillae were observed [46, 53].

According to data about the predominance of Fu papillae on the ventral surface to dorsal surface on the lingual apex we established in all examined ruminants, the number of taste buds on the ventral apex ranged from 900 to 4000 and was 60-95% higher as on the dorsal surface of the apex. Furthermore, that the calculated number of taste buds in the anterior part of the tongue in cattle, European bison, and their hybrid concerning taste buds on all torus surfaces was higher by 60, 46, and 67%, respectively. A characteristic feature observed in Fu papillae on the anterior dorsal part of the *Bison bonasus* hybrid tongue was a significant decrease in the number of taste buds of 1-3 in a single papilla. The calculation showed that in cattle and European bison, as parental species, were around 94%, and almost 82% more taste buds in a single papilla than in their hybrid.

The well-developed system of taste buds noticed in the anterior part of parental species tongues, i.e., apex and body, support data about the high number and density of Fu papillae, especially on the ventral surface of the lingual apex. It also confirms that the anterior part of the tongue is an important functional area in the initial analysis or preselection of food during food intake. On the other hand, a small number of taste buds per single papilla in the anterior dorsal surface of the examined hybrid tongue seems to be inconsistent with these assumptions. However, a large number of Fu papillae on the dorsal surface of the apex and lingual body in the *Bison bonasus* hybrid compensate a phenomenon of a reduced number of taste buds and in final calculations showed that the number of places of taste perception in the hybrid may be comparable to its parental species, and precisely to cattle. On torus, the total number of taste buds was grown from the medial to the posterior part about 3-5 times depending on species and reached 280-1110. Moreover, in European bison and European bison hybrid, the data showed 41 and 30% higher number of taste buds on lateral surfaces than the posterior dorsal part of the torus. In mentioned species, the total number of taste buds on the whole dorsal surface of the torus is smaller than on lateral surfaces.

Functionally, the lingual torus is associated with the process of mastication of food, which was confirmed in our analysis by the smallest total number of taste buds in Fu papillae in the medial part of the torus in all studied ruminants. In some earlier studies, giant Fu papillae in the posterolateral surface of the torus were observed [25, 54–57]. Our results showed that the diameter of Fu papillae on lateral surfaces of the torus was similar or lower than on the other parts of the torus. Still, the total number of taste buds in this area was visibly higher, indicating the importance of lateral surfaces of the torus in taste perception. These observations defined the torus as a part of the tongue significant in both mechanical and sensory functions. Anyway, the perception of the taste of studied ruminants will be a matter for further studies on the number and types of receptors cells of taste buds.

The analysis of 3D models of Fu papillae allowed us to observe for the first time the exact distribution of taste buds on the dorsal surface of Fu papillae. The obtained results are exclusive because we defined the arrangement of taste buds as dispersed manner, but often as diagonal and vertical stripes or clusters in various shapes. In cattle, numerous taste buds were evenly dispersed or formed clusters of taste buds in the middle part of papillae, whereas in European bison, the strips were in the middle part or on borders of Fu papillae. The sparse taste buds in European bison hybrid were positioned most often centrally. If we compare the distribution of taste buds in many parts of the tongue, one observation seems to be exceptional. The taste buds in Fu papillae on the lingual body and on lateral surfaces of lingual torus formed strips mostly arranged perpendicularly to the direction of passage of food along with the tongue. Such tendency of grouping taste buds may allow frontal contact during transportation of food and enhance the taste perception of masticated plant food on the lingual body or during passage grinding food particles in an area between teeth and torus.

The obtained comparative data revealed some species-specific features in the *Bison bonasus* hybrid, which is rare in the case of interspecies ruminant achieved in 1970 in Poland and still occurred in farms. The *Bison bonasus* hybrid called żubroń was crossbred as a farm ruminant due to the desire to establish a new breed of beef cattle adapted to free breeding [58]. Considering data obtained in the *Bison bonasus* hybrid, quite surprising was ca. 3.5 times larger total amount of Fu papillae than in parental species, and the fact that on each part of the *Bison bonasus* hybrid tongue, the number and density of Fu papillae per 1 cm^2^ exceeded values observed in cattle and European bison. However, the protrusion of Fu papillae on each part of tongue was ca. 50% lower papillae then in its parental species. The observations of 3D models of Fu papillae also indicated a small amount or a lack of taste buds in Fu papillae on the lingual apex or the presence of only balloon-like CTCs in Fu papillae on the lingual torus. Our research has shown an interesting similarity of many traits of Fu papillae between the *Bison bonasus* hybrid and the maternal species i.e., domesticated cattle. These common features are almost equal number of taste buds in cattle and the hybrid as number and density of Fu papillae on the particular parts of torus as well the thickness of the stratified squamous epithelium on Fu papillae. These listed features in *Bison bonasus* hybrid and earlier described anatomical data might be considered as species-specific, which could be functional implications and/or are consequences arising after hybridization.

## Conclusions

Quantitative and qualitative data from microscopic observation and computer-aided analysis of 3D models of Fu papillae in three species of ruminants provided new data about the variety of the distribution and microscopic features of Fu papillae over the surface of the tongues. In this respect, two regions of the tongue, i.e., the ventral surface of the lingual apex and posterolateral surfaces of the lingual torus, were designated. The regional differences obtained an evaluation of amount, the density of Fu papillae, and taste buds stay with feeding habits and kind of forage typical for domesticated farm species like cattle and wild living European bison. The characteristic of taste buds system in terms as a number, as well its different arrangement indicated an efficient system of receptions of taste in cattle and less developed complex system in European bison hybrid, which may correlate with evolutionary changes in frame of domestication. The 3D models of Fu papillae in all studied species of ruminants allowed to describe firstly three types of CTC of Fu papillae,

As an innovative method, the three-dimensional analysis proved to be a comprehensive tool for evaluating detailed structures of Fu papillae, i.e., the papillae outline, the shape of CTC, and distribution and number of taste buds. Based on numerous results obtained from 3D models of Fu papillae, we state that the application of this modern visualization technique is the most suitable tool to describe gustatory papillae with taste buds system in mammals and could be helpful in normal, pathological, and clinical assessments.

## Materials and methods

The observations and 3D models of Fu papillae were conducted on one tongue of *Bison bonasus* hybrid and its parental species: cattle and European bison. The cattle tongue was obtained in a cattle slaughterhouse near Poznań, the European bison was from Poznań zoological garden, while the hybrid was from the farm in Karolew, near Poznań in Poland. Each tongue was fixed in 10% neutral formaldehyde. A total of 41 Fu papillae were dissected from the dorsal and ventral surface of the apex, body of the tongue, and for first time from three areas i.e., dorsal, posterior, and lateral surfaces of torus of tongue for three-dimensional analysis.

Tissues dissected for histology and 3D reconstructions proceeded into paraplast. Embedded tissues were serially cut on a microtome (Leica RM 2055) into 8 μm slices. All slides were dye using Masson-Goldner method and closed using synthetic resin DePex. Each slide was observed and documented using a light microscope Axioskop 2 plus (Zeiss) with digital camera Gryphax. For each section, magnification × 1.25 was used. A record of lost or unsuitable slices within serial slides was conducted. Depend on the size of Fu papilla 100-300 slices per papilla were used for reconstruction. In order to create 3D reconstructions of Fu papillae, their CTCs, and taste buds, a conversion to a single channel (green channel), an alignment, a recalculation into uniform coordinates, a segmentation, a resampling in X, Y, and Z direction, a surface generation and adjustments steps in professional computer software, called Amira were conducted (Thermo Fisher Scientific, ver.2020). Moreover, to better visualization of 3D models, one animation of the Fu papilla from the ventral surface of the apex in cattle also in Amira were prepared (Additional file 1).

Morphometric analysis conducted on 2 – 3 Fu papillae dissected from particular parts of examined ruminants tongues. Depended on its size, 8-10 cross-sections from the medial part of the papilla were selected. Furthermore, on each perpendicular cross-section, 3-5 measurements of the height of Fu papillae and its stratified squamous epithelium, the diameter of Fu papillae, the height of interpapillary epithelium, and the diameter of taste buds were taken. These data were obtained using MultiScan computer system v.18.03 (CSS, Warsaw, Poland). Average values of obtained data with its graphical analysis were estimated in Microsoft Excel (2010). The number and density of Fu papillae were established from macroscopic pictures of particular parts of examined tongues, whereas the number and density of taste buds were obtained from 3D models of Fu papillae.

Anatomical nomenclature of the structures of the tongue was taken from Nomina Anatomica Veterinaria (2017) [59].

## Supplementary Information

**Additional file 1.**

## Data Availability

All data analyzed during this study are included in this published article. Any additional data are available upon request to the corresponding author.

## Abbreviations

CTC: Connective tissue core
Fu papillae: Fungiform papillae
Fo papillae: Foliate papillae
LM: Light microscopy
SEM: Scanning electron microscopy
Vp papillae: Vallate papillae
